# Cytotoxicity of Nanoparticles Contained in Food on Intestinal Cells and the Gut Microbiota

**DOI:** 10.3390/ijms17040509

**Published:** 2016-04-06

**Authors:** Esther E. Fröhlich, Eleonore Fröhlich

**Affiliations:** 1 Institute of Experimental and Clinical Pharmacology, Medical University of Graz, Universitätsplatz 4, Graz A-8010, Austria; esther.froehlich@medunigraz.at; 2 Center for Medical Research, Medical University of Graz, Stiftingtalstr. 24, Graz A-8010, Austria

**Keywords:** silver, zinc oxide, nanotoxicology, cytotoxicity, antimicrobial effects

## Abstract

Toxicity of nanoparticles (NPs) upon oral exposure has been studied in animals using physiological changes, behavior, histology, and blood analysis for evaluation. The effects recorded include the combination of the action on cells of the exposed animal and the reaction of the microorganisms that populate the external and internal surfaces of the body. The importance of these microorganisms, collectively termed as microbiota, for the health of the host has been widely recognized. They may also influence toxicity of NPs but these effects are difficult to differentiate from toxicity on cells of the gastrointestinal tract. To estimate the likelihood of preferential damage of the microbiota by NPs the relative sensitivity of enterocytes and bacteria was compared. For this comparison NPs with antimicrobial action present in consumer products were chosen. The comparison of cytotoxicity with *Escherichia coli* as representative for intestinal bacteria and on gastrointestinal cells revealed that silver NPs damaged bacteria at lower concentrations than enterocytes, while the opposite was true for zinc oxide NPs. These results indicate that silver NPs may cause adverse effects by selectively affecting the gut microbiota. Fecal transplantation from NP-exposed animals to unexposed ones offers the possibility to verify this hypothesis.

## 1. Introduction

Contact of nanoparticles (NPs) with the gastrointestinal microbiota occurs mainly via ingestion of solid food, water, cosmetics, and personal care products. In the latter NPs are included as active ingredients to inhibit, for instance, biofilm formation on teeth. However, an action of the NPs in these products on the human body is not intended. The broad use of NPs and its accumulation in the environment warrants studies not solely focusing on acute toxicity in human cells. On the other hand, it is known that microbiota have a pronounced influence on human health [1,2,3]. Due to the close interaction between microbiota and the human organism, it might be hypothesized that effects of NPs on inherent or intestinal bacteria could have an effect on human health.

The human body offers multiple sites for microbiota, like the oral cavity or the gastrointestinal tract. The latter harbors the largest community of bacterial members and has attracted interest from diverse areas of research. The intestinal microbiota comprises bacteria, fungi, viruses and arachaea, which form a complex ecosystem and live in a close relationship with the host. The environment, genetics, diet, and antibiotics shape and alter the microbiota and further influence the interaction between the microbiota and the host. These influences can lead to microbial imbalance (dysbiosis) and may promote susceptibility to diseases. The intestinal microbiota fulfills multiple functions for the host including the processing of otherwise indigestible food compounds, synthesis of vitamins, colonization resistance, regulation of the metabolism, and development of the immune system [4,5]. The latter, in particular, is thought to play an important role when it comes to various diseases including, but not limited to, autoimmune diseases, allergies, inflammatory bowel disease, intestinal infections, obesity, and even neurodevelopmental disorders [4,6,7]. The presence of an established microbiota is of particular importance for normal immune responses, as mice raised under germ-free conditions have been shown to possess an underdeveloped immune system [7,8]. Furthermore, several studies have demonstrated that the microbiota shaped in the first years has an immense impact on an individual’s health status later on in life. Children born by caesarian section have been shown to be at greater risk of developing diseases like asthma, celiac disease or obesity [6]. Due to the growing appreciation of the importance of the microbiota with regard to diseases and the great achievements in sequencing methods and databases, the microbiota has even been suggested as a diagnostic and prognostic biomarker [1,6]. As each individual harbors its own microbiota profile, much like a (personal) fingerprint, the use of microbiota as biomarker would be a big achievement in the field of personalized medicine [1]. The application of the microbiota as therapeutic has already started. Various studies have shown that in patients with recurrent *Clostridium difficile* (*C. difficile*) infection fecal microbiota transplantation—a procedure in which fecal matter, or stool, is collected from a tested donor, mixed with a saline or other solution, strained, and placed in the patient by colonoscopy, endoscopy, sigmoidoscopy, or enema—was effective and has emerged as a promising new treatment for *C. difficile* infection. However, the choice of a donor microbiota needs to be made wisely and further development and research in this field are still needed [4,9].

*In vitro* and *in vivo* studies have usually focused on direct toxic effects of NPs on the exposed cells and organisms, while few studies have investigated potential effects of NPs on the oro-gastrointestinal microbiota of the host. The effects of zinc oxide (ZnO) and cerium oxide (CeO_2_) NPs at 0.01 µg/L and of 3 mg/L titanium oxide (TiO_2_) on microbiota isolates from one healthy donor cultured in a custom colon reactor indicated that NPs affected short fatty acid production, hydrophobicity, sugar content of the extracellular matrix and electrophoretic mobility [10]. Other studies have focused on the composition of the microbiota and have found that silver (Ag) NPs of 14 nm did not alter the ratio of Bacteroides to Firmicutes after oral exposure of rats [11]. The lack of obvious changes in the microbiota composition has also been reported after exposure of mice to 20 and 110 nm Ag NPs [12]. Williams *et al.*, on the other hand, have detected size- and dose-dependent changes in ileal-mucosal microbial populations after oral gavage of rats with 10, 75 and 110 nm Ag NPs [13]. After treatment with 10 nm Ag NPs greater proportions of Firmicutes phyla, along with a decrease in the Lactobacillus genus were observed. In the absence of morphological damage to enterocytes, the population of lactic acid bacteria was increased in the guts of Japanese quail that received colloidal 25 mg/kg Ag NPs in their drinking water [14]. Exposure to 110 nm Ag NPs caused a decrease in Firmicutes at the highest concentration of 36 mg/kg. 60–100 nm Ag NPs also reduced coliforms in the gut microbiota of weaning pigs [15]. When using *in vitro* exposures of the porcine microbiota samples effects were even more pronounced; coliforms were markedly and lactobacilli slightly reduced. In synthetic stool mixtures of 33 different isolates from a healthy human donor polyvinylpyrrolidone-capped 10 nm Ag NPs increased the abundance of *Escherichia coli* (*E. coli*) [16]. These changes were observed at concentrations of ≥100 mg/L. Differences in particle concentrations (9–40 mg/kg) as well as in sizes (10–110 nm) could serve as explanation for the (above-mentioned) contradictory findings. Effects of larger Ag NPs could be explained by closer contact with the bacteria, while smaller NPs could be absorbed by the intestinal tract. Furthermore, adult rats, mice, humans, quails, and weaning pigs differ in composition and stability of the gut microbiota. Different study results can be explained by the fact that the samples used for microbiome analysis may originate from luminal content or from gut tissue. Moreover, the site of specimen collection influences the results, as the number and composition of gut microbiota changes depending on the location in the intestinal tract. In addition to that, the choice of method for the analysis of the microbiota generates method-specific results and may lead to additional bias [17].

The studies mentioned above do not give any indication of changes in organ histology, blood count, and clinical chemistry of the exposed animals. Effects on microbiota, on the other hand, could explain some effects of NPs observed in *in vivo* studies. Oral exposure of rats to ZnO NPs induced not only liver damage but also behavioral changes in the treated animals [18]. Since behavioral changes due to alterations of the gut microbiota have been reported [19,20,21], it might be hypothesized that ZnO caused the behavioral effects by affecting the gut microbiota.

Due to the conflicting results regarding the effects of Ag NPs on the microbiota in animal experiments, the limitations of the methodologies employed so far to assess effects on microbiota, and the limitations of rodent studies for the human microbiota, another approach will be used in this paper in order to evaluate the possibility that NPs act selectively on the gut microbiota. As most research on the microbiota has focused on the role of the bacteria, this review will also concentrate on the bacterial fraction in the gut, and compare the sensitivity of bacteria and intestinal cells to NPs. The selection of the NPs in this review is based on the hypothesis that selective damage of microbiota would be most likely for NPs that are taken up by the oral route and possess antimicrobial activity.

## 2. Oral Ingestion of Nanoparticles

Ag, silica (SiO_2_), TiO_2_, and ZnO NPs are most relevant for oral ingestion because they are added as ingredients to food and contained in health care products. Since 2007, the use of NPs in food and beverages has increased from 64 to 72 products. TiO_2_, ZnO, SiO_2_ are produced in the highest amounts, while Ag NPs are used in the highest number of products [22].

### 2.1. Estimated Amounts of Daily Intake

Ag NPs are used in food packaging, added as antimicrobial agent (E174) to beef, and serve in alginate gel coatings of carrots and asparagus to prevent water loss [23]. Ag NPs can also leach and migrate from plastic bags and reusable food containers. Leaching of Ag from reusable food containers to food simulants in water was 5 ng/cm^2^ over 10 days, polyethylene bags released 10 ng/cm^2^ after 10 days, and 34 ng/cm^2^ of Ag were released after 3 use cycles from food storage containers [24,25,26]. Migration of Ag NPs from plastic food containers amounted to 1.66–31.46 ng/cm^2^ [27]. In addition, bioaccumulation in plants and fungi by Ag content of wastewater, incorporation into sewage sludge and spreading on agricultural fields as well as accumulation within food fish results in human exposure to Ag NPs [28,29]. Based only on food intake, daily Ag consumption is estimated to amount to 20–80 µg/day [30]. SiO_2_ NPs of different composition are labelled as E551, E554, E556, or E559, and used for instance as an anti-caking agent. The amount ingested daily is estimated to be 1.8 mg/kg (around 126 mg/day for a 70 kg person) [31]. The Scientific Committee on Food of the European Food Safety Authority has estimated the daily intake of SiO_2_ at 20–50 mg for a 60 kg person [32]. TiO_2_, gold (Au), platinum (Pt) and ZnO NPs are ingredients of sunscreens and toothpastes [33]. Highest concentrations of TiO_2_ NPs (E171), however, have been found in sweets [34]. Chewing gums and cookies contain around 1–5 µg/mg of E171 and these authors estimated the daily intake to reach 0.45 mg/kg for an adult (around 31.5 mg/day for a 70 kg person) and 1 mg/kg for children. Lomer *et al.* indicated daily ingestions of 2.5 mg for a 70 kg person in one study and 5.9 mg in another one [35,36]. Daily intake amounts estimated by Powell *et al.* [37] were at 5 mg and by Shi *et al.* at 300–400 µg [38]. Based on survey data on daily food intake and fluid consumption rough estimates can be made of approximate concentrations in the gastrointestinal tract. The National Diet & Nutrition Survey reported 449.7 g of solid food (protein, carbohydrates, and fat) for men and 328.1 g for women [39]. Average fluid intake was at 1.98 L/day with considerable variations across countries (lowest: 1.5 L, Japan; highest: 2.47 L, Germany; [40]). Under the assumption that a volume of 2.5 L of food (solid and fluids) is ingested, and based on the highest and lowest daily ingestions of the respective NPs that have been published, the following concentrations can be estimated: 0.008–0.032 µg/mL Ag NP, 9.3–50.4 µg/mL SiO_2_ and 0.12–12.6 µg/mL TiO_2_ NPs. ZnO NPs are included in nutritional supplements, such as multivitamins, and may be released from food packaging [41]. ZnO NPs were also detected in freshwater snails, showing that these animals accumulated ZnO particles present in water [42]. Estimation of oral ingestion of ZnO NPs is complicated because ZnO ingestion may occur in addition to accidental uptake of health care products and oral uptake of food also through nutritional supplements. Dietary zinc deficiency is a global health problem and a dietary intake of 5–20 mg/person/day of zinc is recommended by the European Commission [32]. Zinc is essential for cells and contained in a variety of proteins (transcription factors, enzymes, *etc.*). It also plays an important role in bacterial defense because secretion of zinc by mucosal surfaces makes bacteria more sensitive to immune cell killing [43]. Accidental uptake of ZnO NPs might cause zinc levels to increase into the toxic range of >50 mg/person/day [32].

To estimate the effects of NPs in food on gut microbiota and intestinal cells, the fact that the particles have more direct contact with bacteria in the lumen than with epithelial cells of the gastrointestinal tract also needs to be taken into account. The epithelial cells of the oro-gastrointestinal tract are covered by a mucus layer that consists of a firmly and a loosely adherent layer and can reach a total thickness of up to 1000 µm, which produces a strong barrier preventing both bacteria and NPs from penetrating cells [44]. Mucus restricts cellular access of NPs both by bonding to mucus fibers through ionic and hydrophobic interactions and by size filtering (for more details see for instance [45]). Residence time of food is shortest in the stomach (3–5 h) and longest in the large intestine (20–30 h) [46]. Residence time in the gastrointestinal tract and thickness of the mucus layer explain why the absorption of 500 nm TiO_2_ particles in the stomach is lowest (0.06%) and highest in the large intestine (4%) [47].

### 2.2. Changes of Nanoparticle Properties in the Gastrointestinal Tract

Physicochemical parameters, size and surface properties are strongly influenced by contact with biological fluids [48]. For oral ingestion, mechanical forces and the prominent pH changes along the oro-gastrointestinal tract need to be considered as well. In the stomach, contractions of up to 150 mm Hg have been measured, but effects on NP agglomeration and aggregation are largely unknown [49]. Changes of pH along the oro-gastrointestinal tract are prominent in the fasted state, but usually buffered to a range of pH 2–6 in the presence of food. A low pH can increase dissolution of particles and enzymes in the digestive fluids can induce denudation of particles. Ag NPs show agglomeration in synthetic gastric fluid by partial dissolution and release of Ag^+^ [50,51]. Based on these findings particle growth was described as partial dissolution of Ag particles in the acidic environment and formation of AgCl on the particles’ surface by Ag^+^ in combination with Cl^−^ released from the environment. The influence of dissolution appeared to be more pronounced for smaller particles as they agglomerated to a higher degree [52]. Less conclusive data has been obtained concerning the role of particle coating because polyvinylpyrrolidone-coated Ag particles from various sources behaved differently. Gastric fluid induced rapid dissolution of ZnO NPs [53], while NPs with a lower solubility in acid solutions, such as SiO_2_ NPs, agglomerated in gastric fluid [54]. Stabilization of NPs in food products reduced the extent of changes by oro-gastrointestinal fluids and SiO_2_ NPs integrated in food products deaggregated again after sequential treatment with saliva, gastric juice, and intestinal fluid. Several reports have shown the effect of the food matrix on dispersion and stability; SiO_2_ NPs were better dispersed in low fat coffee creamer than in water, agglomerated in saliva and deaggregated in gastric fluid containing digestive enzymes [52]. SiO_2_ NPs (E551) in coffee, soup, and pancake were nanosized to 30%, 13%, and 5% before and to 80%, 15%, and 15% after subsequent incubation with artificial saliva, gastric juice, and duodenal juice + bile [55]. Furthermore, binding of macromolecules affects the biological action of NPs. The composition of the particle coating (commonly termed as “protein corona”) differs according to the composition of the surrounding media [48]. In the gastrointestinal tract, the protein corona consists of bile salts and proteins. The effects of fluids of the digestive tract on the biological effects of these NPs in intestinal cells have been reported differently. Digestion of Ag NPs with food compounds did not change uptake by Caco-2 cells, while digestion in the absence of food decreased cellular uptake to 60% [56]. Treatment with digestive solutions reduced the potential to generate reactive oxygen species of SiO_2_ NPs without affecting cytotoxicity [57]. Adhesion to enterocytes by the presence of a protein corona was influenced in such a way that coating with bovine serum albumin and casein reduced adhesion of the particles to Caco-2 cells, while coating with meat extract had no effect on cell adhesion of 20, 100, and 200 nm polystyrene particles [58]. Incubation in murine intestinal fluid, however, increased adherence of 20 and 200 nm particles to Caco-2 cells.

## 3. Antimicrobial Activity of Nanoparticles

Due to their antimicrobial activity, aluminum oxide (Al_2_O_3_), Ag, copper oxide (CuO), and ZnO NPs are the particles most likely to affect the gut microbiota [59]. Antimicrobial activity of TiO_2_ NPs was linked to photoactivation and effects were recorded only after illumination [60,61,62]. Naked SiO_2_ NPs did not possess prominent antimicrobial action, while SiO_2_ NPs grafted with antibacterial agents, such as antibacterial polymers, quaternary ammonium compounds, and antimicrobial tricosan displayed antibacterial properties [63]. Antimicrobial effects caused by Al_2_O_3_ and CuO NPs are not relevant for human oral exposure because these particles are only contained in products that do not have a high probability of being ingested, such as abrasives and scratch-proof car paints (Al_2_O_3_) or antimicrobial coatings of pillowcases and socks (CuO) [64].

Therefore, the combination of exposure by the oral route and antimicrobial action restricts the candidates for potential damage of the microbiota to Ag and ZnO NPs.

## 4. Effects of Nanoparticles on Prokaryotic and Eukaryotic Cells

**Figure 1:**
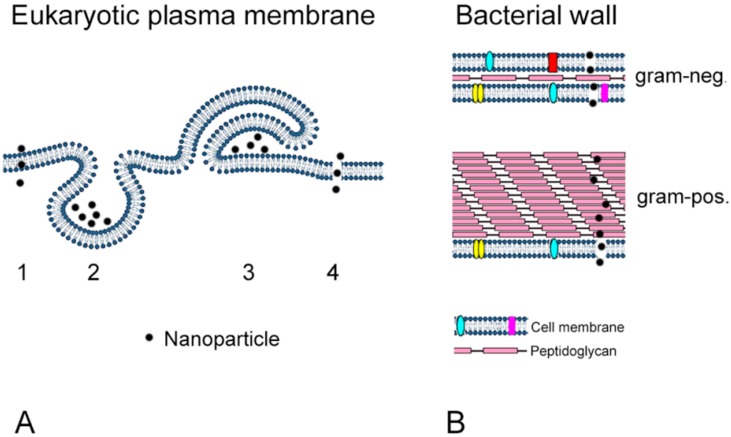
Uptake of NPs by mammalian cells (**A**) and by bacteria (**B**). (**A**) NPs can cross the plasma membrane by diffusion (1), endocytotic uptake (2, 3), and disruption of membrane integrity (4). Endocytosis can occur either by invagination of the membrane (clathrin, caveolin, clathrin- and caveolin-independent routes, 2) or by evagination (macropinocytosis, 3); (**B**) NPs permeate the bacterial wall of gram-negative bacteria, consisting of an inner membrane, a peptidoglycan layer and an outer membrane, and the wall of gram-positive bacteria (membrane + several peptidoglycan layers) by membrane disruption. Membrane proteins are indicated in different colors in the bacterial cell membrane but were not shown in the mammalian plasma membrane.

Toxicity of NPs to bacteria and mammalian cells is linked to the increased reactivity of these particles due to their large surface. However, cellular action of NPs differs between prokaryotic and eukaryotic cells due to their different composition and morphology. One important point is the absence of active uptake mechanisms in bacteria, except planctomycete *Gemmata obscuriglobus* [65]. The plasma membrane of mammalian cells measures 7.5 nm [66]. Uptake into the cells can occur either by diffusion or by active (endosomal) uptake mechanisms. These uptake routes are globally classified as clathrin-dependent (clathrin-mediated) and clathrin-independent. The latter consists of caveolin, clathrin- and caveolin-independent routes, and macropinocytotsis (Figure 1A). Clathrin- and caveolin-independent routes include Arf6-, flotillin-, Cdc42- and RhoA-dependent uptake [67]. For a more detailed summary of the uptake routes for NPs, the reader is referred to reviews dedicated to this topic, for example [67,68,69]. The cell wall of bacteria differs between gram-positive and gram-negative bacteria. Gram-positive bacteria possess one cytoplasmic membrane and one thick peptidoglycan layer of an entire thickness between 20 and 80 nm, while the 5–10 nm thick cell wall of gram-negative bacteria consists of two cell membranes and one thin peptidoglycan layer [70] (Figure 1B).

**Figure 2:**
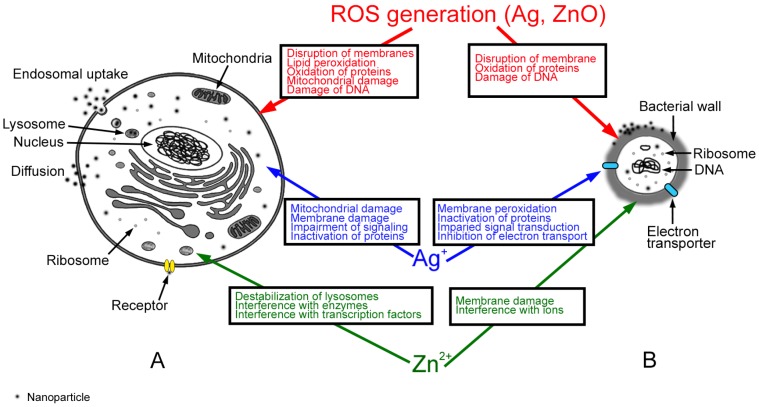
Effects of Ag and ZnO NPs in eukaryotic (**A**) *versus* prokaryotic (**B**) cells with mechanisms (ROS generation, action of Ag and Zn ions) indicated by different colors. NPs may enter mammalian cells either by endosomal uptake or by diffusion. For bacterial cells, the only uptake mechanism is diffusion across the bacterial wall. Target organelles for NP effects, such as mitochondria, lysosomes and nucleus, are indicated. Ribosomes are essential components of mammalian and bacterial cells; they are in a similar size range as NPs. ROS, reactive oxygen species.

Additional differences between bacterial and mammalian cells include the around 50 times larger cell size of mammalian cells, the presence of membranes around the nucleus and of membrane-enclosed organelles (endosomes, lysosomes, autophagosomes, mitochondria, peroxisomes, *etc.*), and the cytoskeleton (Figure 2A). Bacteria, on the other hand, have a cell wall instead of a plasma membrane and a circuit chromosome devoid of histones [71] (Figure 2B). The majority of NPs that enter mammalian cells by active mechanisms are transported to lysosomes. There, low-biodegradable NPs can accumulate, metal ions can be released and increase cytotoxicity [72]. When NPs reach the cytoplasm by diffusion across the plasma membrane, the release of metal ions and cytotoxicity are lower [73]. The lack of endocytosis in bacteria has the important consequence that NPs enter bacteria only by destroying the bacterial wall and cell membrane [74] (Figure 2B). The NPs anchor to the bacterial wall and penetrate it causing structural changes. Ag NPs probably bind to thiol groups of membrane proteins. Electrostatic attraction to the cell membrane, however, is less likely because both membrane and Ag NPs are negatively charged. Another option is the formation of irregular pits at the bacterial surface leading to NP accumulation followed by progressive release of lipopolysaccharides and membrane proteins facilitating uptake by bacteria [75].

### 4.1. Mechanisms of Ag Nanoparticle (NP) Action

Cytotoxicity data in mammalian cells is usually determined after 24 h of exposure, which is a physiologically relevant time point because typical gastrointestinal transit times in healthy individuals are 22–26 h for ingestion in the morning and 38 h for ingestion in the afternoon [76]. Cytotoxic effects of Ag NPs on intestinal cells are mainly due to the release of Ag^+^ ions (Figure 2A), but it is often difficult to differentiate between effects caused by particles and those caused by ions because many studies did not use silver solutions as controls. On the other hand, ions and particles were shown to cause similar effects, namely damaging the plasma membrane and mitochondria. Mitochondrial damage may subsequently lead to the generation of reactive oxygen species (ROS) resulting in lipid peroxidation, oxidation of proteins and DNA damage [77]. Binding of Ag^+^ ions to proteins may cause loss of function and impair cell signaling. The comparison of different cell lines demonstrated that the intestinal Caco-2 cells reacted less sensitively to Ag NP cytotoxicity than liver HepG2 cells. 20 nm Ag NPs caused mitochondrial damage in Caco-2 cells in the absence of ROS generation [78]. Studies using 10, 20, 40, 60, and 100 nm Ag NPs on LoVo colon cancer cells confirmed the damaging action of Ag NPs on mitochondria [79]. However, these authors showed that ROS generation was linked to mitochondrial dysfunction.

Antimicrobial action of metal and metal oxide NPs is often linked to the release of ions that interact with bacterial membranes by electrostatic interaction, increase of membrane permeability, and peroxidation of polyunsaturated membrane phospholipids [59]. Furthermore, ROS generated by NPs can oxidize membranes and proteins resulting in impaired respiration and cell division. Ag^+^ ions released by NPs can disrupt ATP production and DNA replication and cause ROS generation and direct membrane damage (Figure 2B). Oxidative damage of membrane proteins and DNA plays a major role in the antibacterial action of Ag NPs. Ag^+^ ions affected the bacterial metabolism by inactivating proteins and subsequently impairing signal transduction and respiratory chain function [80]. Oxidative dissolution of Ag NPs takes place at a sufficiently high concentration of H^+^ in mitochondria or in the presence of oxygen. Effects of Ag^+^ ions are concentration-dependent; at micromolar concentration, they interact with NADH dehydrogenase uncoupling ATP synthesis. They bind to membrane transport proteins, which results in proton leakage, and they inhibit phosphate uptake resulting in efflux of intracellular phosphate [75]. Millimolar concentrations of Ag^+^ ions lead to cytoplasm shrinkage, detachment of the cell wall membrane, destruction of the peptidoglycan cell wall, denaturation of ribosomes, DNA condensation with inhibition of DNA synthesis, and lysis of the cell membrane [80]. Programmed cell death, frequently induced by NPs in mammalian cells, may also be caused by Ag NPs in bacteria [81].

The thicker bacterial wall of gram-positive bacteria is supposed to better protect against toxicity of Ag NPs, as gram-negative bacteria were more sensitive to toxicity of Ag NPs than gram-positive ones [82]. The difference of ZnO particle toxicity between gram-positive and gram-negative bacteria, however, was not pronounced [83]. The greater sensitivity of gram-negative bacteria may be explained not only by the thinner cell wall but also by the lower amount of the negatively charged peptidoglycan which can trap the positively charged silver ions [84]. Bacteria growing under aerobic conditions reacted more sensitive to Ag NPs than anaerobe bacteria [82]. It was speculated that higher sensitivity was due to higher oxidative dissolution of Ag particles.

### 4.2. Mechanisms of ZnO NP Action

Similar to Ag NPs, ZnO NPs act cytotoxic by particle effects in combination with ion release. Release of Zn^2+^ ions disrupted homeostasis of gastrointestinal cells and interfered with the activity of Zn-containing enzymes and transcription factors [85] (Figure 2A). Cellular contact of the particles appeared to be more important than extracellular concentration of Zn^2+^ ions [85] suggesting that Zn^2+^ ions were mainly released inside the cells. The acidic pH of the lysosomes promoted the release, which was accompanied by cellular oxidative stress and mitochondrial damage. Cytotoxicity of ZnO NPs in sizes of 8–70 nm has been reported in several intestinal (RKO, Caco-2, LoVo, C2BBe1) cell lines [86,87,88,89,90]. Moreover, mitochondrial damage has been reported quite consistently, while reports are conflicting regarding ROS generation. Indication of oxidative stress by decreased cellular GSH levels and depolarization of the inner mitochondrial membrane has, for instance, been reported in LoVo cells exposed to 50–70 nm ZnO NPs. One explanation for not detecting ROS in all studies might be that zinc acts as antioxidant and could interfere with the oxidation of the commonly used detector dye dihydrodichlorofluorescein [91].

ZnO NP-induced antibacterial effects were caused by a combination of Zn^2+^ ions, induction of ROS and mechanical forces (electrostatic and abrasive forces) [92] (Figure 2B). Interaction with intracellular ions was specific for ZnO NPs and was not observed for Ag NPs. The underlying mechanisms were membrane damage, and oxidation of proteins as well as DNA resulting mainly from ROS generation [93]. Furthermore, inhibition of sugar transport by Zn^2+^ ions, and displacement of Mg^2+^, which is essential for biofilm formation, occurred [94]. Abrasive forces of edges, corners, spatial configurations, and defects of the particle surface caused physical damage of the bacterial wall [92]. As ZnO particles in water possess a positive surface charge, they can inhibit bacterial growth by electrostatic interaction with the cell membrane.

## 5. Comparison between Intestinal Cells and Bacterial Toxicity

In order to compare the sensitivity of mammalian cells and bacteria to Ag and ZnO NPs, a common indicator for cytotoxicity is needed. The concentration causing the half-maximal effect, termed *EC_50_* value, is a common indicator for mammalian cytotoxicity and allows for a comparison between different cell types and screening assays. Weight/volume or weight/area is commonly indicated and interconversion of the two values is possible when both volume of the exposure solution and plate format are known. Antimicrobial activity is determined by read-out parameters, which cannot be easily interconverted. The most common methods to monitor bacterial growth include broth dilution followed by colony count (plating of serial culture broth dilutions), agar diffusion method, which is the official method for bacteriostatic activity, and growth curve monitoring at 600 nm [92]. Effects are usually reported as minimal inhibitory concentration (MIC), half-maximal effective concentration (*EC*_50_) values or diameter (in mm) of inhibition zones. MIC is the lowest concentration of an antimicrobial that inhibits the visible growth of a microorganism and *EC*_50_ is the concentration of a compound or a NP that gives half-maximal response *in vitro*. In contrast to the relatively constant incubation conditions in the cytotoxicity screening (37 °C, 24 h), the experimental set up for antibacterial activity differs regarding evaluation time and temperature.

In the screening for antimicrobial activity, specific bacterial strains are included due to their pathophysiological relevance for humans. These bacteria are *E. coli*, *Staphylococcus aureus* (*S. aureus*), *Pseudomonas aeruginosa* (*P. aeruginosa*), *Klebsiella pneumonia* (*K. pneumonia*), *Bacillus subtilis* (*B. subtilis*), and *Salmonella typhi* (*S. typhi*) [95].

*E. coli* was chosen as bacterial representative of the intestinal microbiota for cytotoxicity of NPs. *E. coli* is the most common aerobe in the human gut microbiota and constitutes 0.1%–5% of the bacterial community in the gut [96,97]. Commensal *E. coli* strains usually inhabit the thin mucus layer that lines the gut and interact with further/other members of the intestinal microbiota. Living in a mutual relationship with the host, *E. coli* provides supplemental nutrition and is involved in resistance to pathogen colonization [96,98]. Moreover, Breton *et al.* have recently shown that proteins of gut commensal *E. coli* were even able to influence host appetite [99].

Out of the most often tested intestinal bacteria, *E. coli* has an intermediate sensitivity to NPs. *EC*_50_ values for ZnO NPs differed between 94 mg/L in *S. aureus*, 181 mg/L in *E. coli*, and 936 mg/L in *P. aeruginosa* [83]. Several other studies support the stronger effect of Ag NPs in *E. coli* than in *S. aureus* (Table 1). However, it has also been reported that there are no differences between the above-mentioned bacteria. In summary, *S. aureus* has rarely been shown to react more sensitive to Ag particles than *E. coli* [100]. The generally greater resistance of *S. aureus* against Ag NPs has been explained by the thicker peptidoglycan layer of the bacterial wall of gram-positive bacteria [101] and has not been observed for the antimicrobial action of ZnO NPs. Ag and ZnO NPs have different modes of action and antimicrobial effects of ZnO NPs are due to a smaller extent to dissolved ions and to a higher extent to ROS generation and to mechanical effects [92,93]. This might explain the missing correlation of bacterial wall composition and susceptibility to antimicrobial effect seen for Ag NPs but not for ZnO NPs. Strain differences need to be considered as well, as 10 and 15 nm Ag NPs, for instance, showed higher potency in *E. coli* MTCC433 than in *E. coli* MTCC739 isolates [102].

### 5.1. Antimicrobial Effects

The study by Bondarenko *et al.* [135] summarized the (eco)toxicology of Ag, CuO, and ZnO particles on a wide panel of cells and organisms (bacteria, yeast, algae, nematodes, crustaceans, mammalian cell lines, fish) including data from 5 studies on Ag and 5 studies on ZnO NPs in *E. coli*. The authors found variations in the antibacterial effects of 500 times for Ag NPs and of 16 times for ZnO NPs [135]. The great variation of the effects underscores the importance of standardized testing protocols because not only particle parameters (size, surface charge, *etc.*) but also preparation of the samples, exposure conditions (duration, temperature, *etc.*) and detection method (colony count, agar diffusion, *etc.*) complicate inter-study comparisons. The summary of the 52 studies for Ag NPs listed in Table 1 showed variations in the same order of magnitude. Variations were more prominent for particles >20 nm (0.25–125 µg/mL) than for particles ≤20 nm (0.3–60 µg/mL). For ZnO NPs, variations were 51.1–3100 µg/mL for particles ≤20 nm and 0.1–927 µg/mL for larger particles. Polyvinylpyrrolidone (PVP) and citrate-coated Ag NPs generally caused a more toxic reaction to bacteria than uncoated ones, which could in part be due to the better dispersion of Ag NPs after coating [105]. One study evaluating the effects of PVP-coated 70 nm Ag NPs in *E. coli*, *S. aureus*, mesenchymal stem cells, and peripheral blood mononuclear cells showed *EC*_50_ values of 12.5–50 µg/mL for all cells [136]. Antimicrobial activity of Ag and ZnO NPs was summarized in this review by focusing on oral ingestion of these NPs and the summaries of the 52 studies on Ag NPs and 16 studies on ZnO are displayed in Table 1. Considerable variations between the studies regarding the reported effects on *E. coli* were seen. Several studies suggested a stronger effect of small particles. 5 nm Ag NPs acted antimicrobial to *E. coli* at much lower *EC*_50_ values than 15 and 55 nm particles [82], and *EC*_50_ values were about 10 times lower for 10 nm Ag NPs than for 60 nm Ag NPs (0.25 mg/L *versus* 2 mg/L; [137]). Similarly, MIC values of various biogenic Ag NPs were in the range of 6.75–54 µg/mL when particles were >25 nm and 1.69–13.5 µg/mL at 25 nm [118]. In our comparison, the median of the MIC values was 8.0 µg/mL for particles ≤20 nm (mean ± SD: 12.9 ± 15.2 µg/mL) and 22.5 µg/mL (mean ± SD: 40.2 ± 51.2 µg/mL) for particles >20 nm. From the biological side, a greater sensitivity of gram-negative bacteria to Ag NPs has been reported [82]. Twenty-one studies (Table 1) support this finding when *E. coli* is taken as an example for gram-negative and *S. aureus* as an example for gram-positive bacteria, while 9 studies did not observe such an effect. When other combinations of gram-negative and gram-positive bacteria are analyzed, only 6 *versus* 10 studies are in favor of this theory. Studies on antimicrobial effects of ZnO NPs have demonstrated greater efficacy of 50–70 nm ZnO NPs against *E. coli* and *S. aureus* compared to 100–300 nm particles. One study has furthermore revealed that 12 nm ZnO NPs were more efficient than 25, 88, 142 and 212 nm and another one has shown that they were more potent than 45 and 2000 nm ZnO particles [126,138,139]. In our comparison, the median of the MIC values was 756 µg/mL for particles ≤20 nm (mean ± SD: 1322 ± 1075.7 µg/mL) and 275.9 µg/mL (mean ± SD: 2342.3 ± 6220.9 µg/mL) for particles >20 nm. No differences in the antimicrobial effect of ZnO NPs have been reported between gram-positive and gram-negative bacteria, which is in line with the study results listed in Table 1. In the combination of *E. coli* and *S. aureus*, only 1 *versus* 5 studies has reported a higher sensitivity of *E. coli*. When several gram-negative and gram-positive bacteria are compared, 1 study has reported a higher sensitivity of the gram-negative bacteria, while another one has not. All studies have reported lower *EC*_50_ values in colon cells than effective antimicrobial effects for ZnO NPs.

The variations observed between the studies can be explained by differences in physicochemical properties of the particles, the pre-treatment prior to the application to bacteria, the methods used for evaluation of antimicrobial effects, the exposure conditions (temperature, length of incubation, medium, *etc.*), and the use of bacterial strains (e.g., *E. coli* MTCC433 and *E. coli* MTCC739). Variations for Ag NPs were higher than for ZnO NPs suggesting that the extent of particle dissolution may also play a role. The antimicrobial effect of ZnO NPs is more directly linked to dissolution and concentrations of Zn^2+^ ions [140] because ZnO NPs dissolve much faster than Ag NPs [141].

### 5.2. Adverse Effects of Nanoparticles on Intestinal Cells (Enterocytes)

Cytotoxicity is commonly assessed in such a way that cells cultured on plastic plates are exposed to NPs suspended in cell culture medium. After exposure of usually 24 h to the particles, effects are assessed by determination of cell number, DNA or protein content or enzymatic activity. Values of the exposed cells are indicated related to the control cells, which are set as 100%. The most commonly used assays, such as MTT, MTS, WST series, and XTT determine the activity of cellular dehydrogenases and reductases by conversion of a tetrazolium salt to a colored product. For the assessment of NPs, specific additional controls have to be included because the particles may interfere with a variety of assays [142]. For the assessment of orally ingested NPs, additional refinements, for instance pre-treatment of particles with gastrointestinal fluids, might be suggested. After sequential incubation in artificial saliva gastric juice and intestinal fluid with organic compounds, such as mucin and enzymes, Ag NPs acted slightly less cytotoxic than untreated particles [143]. The application of ZnO NPs in combination with food components (ZnO + fatty acids), on the other hand, increased their cytotoxic effect [144]. To further improve testing, 3D cultures could be used, where cells grow on artificial membranes instead of plastic surfaces and the frequently used Caco-2 cells differentiate into polarized cells with plasmatic extrusions (microvilli) at the apical side [145]. Testing in these models allows for the identification of additional cellular effects, such as action on intercellular junctions and on cell architecture.

Besides cytotoxicity, NPs can also cause other adverse effects on intestinal cells. These effects include induction of inflammation, alterations of the proliferation rate, genotoxicity, and stimulation of oxidative stress response. An increase in cytokine secretion has been observed after exposure of intestinal cell lines to TiO_2_, Ag, ZnO, and SiO_2_ NPs [89,146,147]. The effect of Ag NPs was stronger than that of TiO_2_ and SiO_2_ NPs of the same size [146]. DNA damage was induced by ZnO and SiO_2_ NPs, yet by different mechanisms. While DNA damage by ZnO and SiO_2_ NPs was linked to the generation of reactive oxygen species, no link between oxidative stress and DNA damage has been observed for TiO_2_ NPs [87,148,149]. Caco-2 cells reacted to Ag NPs with increased proliferation followed by a decrease in the proliferation rate [150]. According to the majority of studies, Ag NPs act as inductors of ROS with a decrease in glutathione levels and an activation of stress-response genes, such as Nrf2 and heme oxygenase 1 [151,152]. In contrast to that, Abbott Chalew *et al.* have reported absence of ROS generation for ZnO, Ag, and TiO_2_ NPs [89].

Finally, the intercellular junction and the brush border (microvilli) of enterocytes appear to be targets for the adverse actions of NPs. Ag NPs and TiO_2_ NPs disrupted intercellular tight junctions [89,129], and microvilli of Caco-2 cells collapsed after exposure of Caco-2 cells to TiO_2_ NPs [153]. The brush border of the enteral epithelium was disrupted after exposure to TiO_2_ NPs isolated from chewing gum [154]. These effects on enterocyte physiology may influence the barrier function and nutrient uptake of intestinal cells.

Bondarenko *et al.* have reported considerable variations in the toxic concentrations in their panel of mammalian cells (not including cells of the intestinal barrier); cytotoxicity of Ag NPs varied 275 times and 20 times for ZnO NPs in the different studies [135]. These differences could be due to different growth patterns, cell size, and proliferation rates of the cells, as these parameters have been shown to influence cytotoxicity of NPs [155]. Particularly the growth pattern was linked to cytotoxicity of NPs, and cells growing in suspension were significantly more sensitive than adherent cells. A certain, but much lower, degree of variation has been observed in our comparison which included intestinal cell lines only (Table 1). Colon cells were more resistant to the toxic effect of Ag NPs than the panel of cell lines compared in the study by Bondarenko *et al.*, who has reported a mean *EC*_50_ for a panel of mammalian cells of 11.3 mg/L [135]. In the literature overview (performed) in this review, the lowest *EC*_50_ value has been identified for HT-29 cells (25 µg/mL, [130]). These variations could not be explained by particle size because cytotoxicity of Ag NPs <20 nm in Caco-2 cells was not markedly higher than toxicity of larger particles [89,129,131]. Cytotoxicity of ZnO NPs in the colon cell lines was given in different ways (significant decreases to <80% or 50% viability or as *EC*_50_), but overall reaction was very similar [88,133,134].

To exclude the influence of different particles and different treatment of particle suspensions, it might be better to compare studies evaluating the same particles in bacterial and mammalian cells. Ivask *et al.* compared 10, 20, 40, 60 and 80 nm citrate-coated Ag NPs in *E. coli* and murine fibroblast cultures and identified considerably lower sensitivity of the mammalian cells to Ag NPs [137]. Differences between fibroblasts and bacteria were particularly evident for the 10 nm particles (*EC*_50_ of 0.25 mg/L in *E. coli* and 2 mg/L in fibroblasts), and much lower for the 80 nm particles (*EC*_50_ of 2 mg/L in *E. coli* and 3 mg/L in fibroblasts). Mammalian cells might be less affected than bacterial cells by reduction of the Ag particle size because changes in NP sizes are more relevant for bacteria, as they are in a more similar size range than mammalian cells (e.g., size of *E. coli* is 0.5 µm × 2 µm, while the diameter of Balb/3T3 fibroblasts is 18 µm, [156]).

ZnO NPs in the range between 20 and 90 nm caused cytotoxicity in colon cell lines of around 20 µg/mL [88,134,157], while antibacterial action against *E. coli* was reported at 0.1–3.1 mg/mL [93,123,127]. The greater sensitivity of mammalian cells to ZnO NPs is very likely to be due to the mechanical (abrasive) forces of these particles and the lack of a protective peptidoglycan layer. The finding that direct contact between particles and colon cells is required for cytotoxic action and that cytotoxicity was largely independent from the extracellular concentration of Zn^2+^ ions is in line with this hypothesis [158]. Abrasive forces could be more relevant for mammalian cells because the thickness of the plasma membrane (6–8 nm) has been given as 5 and 10 times lower than the bacterial envelope of gram-negative bacteria (~35 nm) and gram-positive bacteria (~90 nm) respectively [159]. Other references, however, state that the cell wall of gram-negative bacteria is only 5–15 nm thick [70], which argues against an important role of a greater wall thickness as main reason for the high sensitivity of mammalian cells. Apart from the total thickness, the two membrane structures in the wall of the gram-negative bacteria in combination with peptidoglycan could provide better protection against mechanical forces than the single bilayer of the mammalian cells.

The comparison of antibacterial effects and mammalian cytotoxicity in this study focused on *E. coli* and human intestinal cells. The conclusion, however, that ZnO NPs act more toxic on mammalian cells than on bacteria is the same with regard to the comparisons of a broader spectrum of bacteria and mammalian cells [135]. The literature overview by Bondarenko *et al.* identified a higher toxicity of mammalian cells than of bacteria for ZnO NPs (*EC*_50_ of 43 mg/L, mammalian cells *versus* MIC of 500 mg/L, bacteria), while no differences were reported regarding toxicity by Ag NPs with median *EC*_50_ of 11.3 mg/L for mammalian cells and median MIC of 7.1 mg/L for bacteria. In our study, which focused on gastrointestinal exposure to NPs, a higher sensitivity of bacteria to small Ag NPs compared to mammalian cells was observed. In the majority of studies (24 *versus* 6 studies), MIC values for antimicrobial effects were lower than the lowest *EC*_50_ value measured in colon cells (Table 1). The present comparative study was conducted in order to estimate the likelihood of NPs affecting the gut microbiota in the absence of cytotoxicity on cells of the gastrointestinal lining. Due to the higher sensitivity of gram-negative compared to gram-positive bacteria to Ag NPs [82], changes in the microbiota composition are expected in the case of antimicrobial action of NPs. It is therefore surprising that data from exposures of mice and rats to Ag NPs between 14 and 110 nm did not find indications for such changes [11,12]. The latter could be explained by the fact that only low nontoxic levels of Ag NPs reached the gastrointestinal tract. It has been hypothesized that Ag NPs might not reach the large intestine because they are either absorbed from the gastrointestinal tract [15] or dissolved in the low pH of the gastric fluid. Dissolution of Ag NPs in humans is less likely in the fed than in the fasted state because the gastric pH increases from around 1.7 to 6.7 in the fed state [160]. However, differences between fed and fasted states are less pronounced in mice with pH 3.0 (fed) *versus* 4.0 (fasted) and in rats 3.2 (fed) *versus* 3.9 (fasted) [161]. Furthermore, gastric pH in rodents is less acidic than in humans. Ag^+^ ions from dissolved Ag NPs may also form secondary particles due to precipitation [162], but conditions (necessary) to form these precipitates as well as the extent of precipitate formation in the gut are unknown. Conclusions from the effects observed in rodents that apply to the human situation are limited by the fact that rat and human microbiota differ in their composition and that 85% of bacterial genera found in the mouse gut microbiota are not present in the human gut [163]. Such differences are not surprising, since not only the diet but also the pH of the intestine differ between mice, rats and humans (mouse: pH 5.2, rat: pH 6.6, humans: pH 6.6–7.5) [164]. Interpretation is further complicated by the prominent inter-individual differences in the composition of the human gut microbiota and influences of pathologies [1].

## 6. Conclusions

The provided comparison of toxicity in mammalian and bacterial cells aimed to estimate the likelihood of NP effects on the human organism by selective damage of the gut microbiota. The studies identified led to the hypothesis that Ag NPs could cause such effects, as antimicrobial effects have been observed at lower concentrations than cytotoxicity to intestinal cells. Furthermore, the contact of bacteria with NPs is more intense because a thick mucus layer covering the enterocytes restricts access of NPs. It also appears that smaller Ag NPs act more potently than larger particles. The study by Williams *et al.* has shown pronounced effects from 10 nm Ag NPs on cultivable bacteria isolated from rat feces [13]. Given the higher toxicity of ZnO NPs in mammalian cells than in bacteria, selective damage of the gut microbiota by these particles is not expected. Studies on the physiological consequences of NP-induced change in the gut microbiota are difficult to assess. Fecal transplantation from exposed to unexposed rats might be a way to address this effect in a similar manner as it has already been done for the evaluation of antibiotic treatment [165]. This method may circumvent problems in the interpretation of PCR-based analysis and colony-forming unit formation. However, limitations with regard to the transfer of results to the human situation due to differences in species, gastrointestinal pH, and other individual variations, as well as environmental factors, food intake, and other parameters that affect the composition of the gut microbiota, will remain.

## Figures and Tables

**Table 1:** Toxic action of Ag and ZnO NPs on *E. coli* compared to other gram-positive (+) and gram-negative (−) bacteria and on human intestinal cell lines. Stabilizers of the particles are indicated, when mentioned in the respective reference.

Nanoparticle	Size (nm)	Bacterial Strain	Effect	Reference
Ag	3	*E. coli* (−)	MIC: 40 µg/mL	[103]
		*S. aureus* (+)	120 µg/mL	
Ag	8, citrate	*E. coli* (−)	MIC: 8 µg/mL	[104]
Ag	10, PVP	*E. coli* (−)	MIC: 10 µg/mL	[100]
		*S. aureus* (+)	5 µg/mL	
Ag	10.5, PVP	*E. coli* (−)	*EC*_50_: 8.9 mg/L	[105]
		*B. subtilis* (+)	5.2 mg/L	
		*S. aureus* (+)	16.1 mg/L	
		*P. aeruginosa* (−)	0.59 mg/L	
Ag	12.7	*E. coli* (−)	MIC: >10 µg/mL	[106]
Ag	13.4	*E. coli* (−)	MIC: >0.35 µg/L	[107]
		*S. aureus* (+)	>3.56 µg/L	
Ag	10–15	*E. coli* (−)	MIC: 25 µg/mL	[108]
		*S. aureus* (+)	100 µg/mL	
Ag	16	*E. coli* (−)	MIC: 60 mg/L	[109]
Ag	4, <20, biostabilized	*E. coli* (−)	MIC: 2, 0.5 mg/L	[110]
		*B. subtilis* (+)	6, 2 mg/L	
Ag	7–20, biostabilized	*E. coli* (−)	MIC: 6.3 mg/L	[111]
		*B. subtilis* (+)	6.3 mg/L	
		*P. aeruginosa* (−)	6.3 mg/L	
		*S. aureus* (+)	12.5 mg/L	
Ag	20	*E. coli* (−)	Inhib: 20 and 23 mm at 10 µg/mL	[112]
		*S. aureus* (+)		
Ag	21	*E. coli* (−)	MIC: 75 µg/mL	[113]
Ag	5, 7, 10, 15, 20, 30, 50, 63, 85, 100, citrate	*E. coli* (−)	MIC: 20, 20, 30, 30, 40, 50, 80, 90, 90, 110 µg/mL	[102]
		*S. aureus* (+)	70, 70, 80, 100, 90, 100, 130, 160, 180, 200 µg/mL	
Ag	20, 50, 110, citrate	*E. coli* (−)	*EC*_50_: 25, 79, 175 mg/L	[114]
Ag	26	*E. coli* (−)	MIC: 1.69 µg/mL	[115]
		*P. aeruginosa* (−)	3.38 µg/mL	
		*S. aureus* (+)	3.38 µg/mL	
		*K. pneumoniae* (−)	6.75 µg/mL	
Ag	1, 29, 89	*E. coli* (−)	MIC: 6.3, 13, 11.8 mg/L 7.5, 16.7, 33.7 mg/L	[116]
		*S. aureus* (+)		
Ag	30	*E. coli* (−)	MIC: 5–10 µg/mL	[117]
Ag	25, >25	*E. coli* (−)	MIC: 1.69–13.5 µg/mL, 6.75–54 µg/mL	[118]
Ag	20–60, PVP	*E. coli* (−)	MIC: 125 µg/mL	[119]
Ag	40–50	*S. aureus* (+)	Inhib: 15–25 mm at 39.5 µg/mL	[120]
		*B subtilis* (+)		
		*K. pneumoniae* (−)		
		*E. coli* (−)		
Ag	50	*E. coli* (−)	MIC: 0.1 µg/mL	[121]
Ag	55	*E. coli* (−)	MIC: 0.25 µg/mL	[122]
		*S. aureus* (+)		
ZnO	3	*E. coli* (−)	MIC: 3.1 mg/mL	[123]
		*S. aureus* (+)	1.5 mg/mL	
ZnO	8, 11, 13	*E. coli* (−)	MIC: >244 mg/L;	[124]
		*S. aureus* (+)	81.41 mg/L	
ZnO	19	*E. coli* (−)	MIC: 500 mg/L	[125]
		*S. aureus* (+)	1000 mg/L	
		*K. pneumoniae* (−)	500 mg/L	
ZnO	30	*E. coli* (−)	MIC: 0.4 mg/mL	[93]
ZnO	47	*E. coli* (−)	MIC: 400 mg/L	[126]
ZnO	50–70	*E. coli* (−)	*EC*_50_: 115.7 mg/L	[127]
		*B. subtilis* (+)	85.8 mg/L	
		*S. aureus* (+)	>125 mg/L	
ZnO	70	*E. coli* (−)	MIC: 972 mg/L	[128]
**Nanoparticle**	**Size (nm)**	**Intestinal Cells**	**Effect**	**Reference**
Ag	<20	Caco-2/Raji B	*EC*_50_: 40 µg/mL	[129]
Ag	18	HT-29	*EC*_50_: 25 µg/mL	[130]
Ag	20–30	Caco-2	*EC*_50_: >100 µg/mL	[89]
Ag	40–50	HT-29	*EC*_50_ (HT-29): 39.5 µg/mL	[120]
Ag	20, 34, 61, 113	Caco-2/Raji B	Viab. >80% at 50 µg/mL	[131]
Ag	1–100	HCT116	sign. decr. viab.: 50 µg/mL	[132]
ZnO	20–60	RKO	30% viab. at 30 µg/mL	[133]
ZnO	26, 62, 90	Caco-2	*EC*_50_: 15.6, 22.9, 18.6 µg/mL	[134]
ZnO	50–70	LoVo	sign. viab. decr. at 10 µg/mL	[88]

*E. coli*: Escherichia coli; *B. subtilis*: Bacillus subtilis; *P. aeruginosa*: Pseudomonas aeruginosa; *S. aureus*: Staphylococcus aureus; *K. pneumoniae*: Klebsiella pneumoniae; decr.: Decrease; *EC*_50_: Effective dose; inhib: Inhibition; sign.: Significant; MIC: Minimal inhibitory concentration; PVP: polyvinylpyrrolidone; viab: Viability.
